# The insect-specific Palm Creek virus modulates West Nile virus infection in and transmission by Australian mosquitoes

**DOI:** 10.1186/s13071-016-1683-2

**Published:** 2016-07-25

**Authors:** Sonja Hall-Mendelin, Breeanna J. McLean, Helle Bielefeldt-Ohmann, Jody Hobson-Peters, Roy A. Hall, Andrew F. van den Hurk

**Affiliations:** 1 Public Health Virology, Forensic and Scientific Services, Department of Health, Queensland Government, PO Box 594, Archerfield, 4108 QLD Australia; 2 Australian Infectious Diseases Research Centre, School of Chemistry and Molecular Biosciences, The University of Queensland, St Lucia, 4072 QLD Australia; 3 School of Veterinary Science, The University of Queensland, Gatton Campus, Gatton, 4343 QLD Australia

**Keywords:** Insect-specific flavivirus, Palm Creek virus, West Nile virus, *Culex annulirostris*, *Aedes aegypti*, *Aedes vigilax*

## Abstract

**Background:**

Insect-specific viruses do not replicate in vertebrate cells, but persist in mosquito populations and are highly prevalent in nature. These viruses may naturally regulate the transmission of pathogenic vertebrate-infecting arboviruses in co-infected mosquitoes. Following the isolation of the first Australian insect-specific flavivirus (ISF), Palm Creek virus (PCV), we investigated routes of infection and transmission of this virus in key Australian arbovirus vectors and its impact on replication and transmission of West Nile virus (WNV).

**Methods:**

*Culex annulirostris*, *Aedes aegypti* and *Aedes vigilax* were exposed to PCV, and infection, replication and transmission rates in individual mosquitoes determined. To test whether the virus could be transmitted vertically, progeny reared from eggs oviposited by PCV-inoculated *Cx. annulirostris* were analysed for the presence of PCV*.* To assess whether prior infection of mosquitoes with PCV could also suppress the transmission of pathogenic flaviviruses, PCV positive or negative *Cx. annulirostris* were subsequently exposed to WNV.

**Results:**

No PCV-infected *Cx. annulirostris* were detected 16 days after feeding on an infectious blood meal. However, when intrathoracically inoculated with PCV, *Cx. annulirostris* infection rates were 100 %. Similar rates of infection were observed in *Ae. aegypti* (100 %) and *Ae. vigilax* (95 %). Notably, PCV was not detected in any saliva expectorates collected from any of these species. PCV was not detected in 1038 progeny reared from 59 PCV-infected *Cx. annulirostris*. After feeding on a blood meal containing 10^7^ infectious units of WNV, significantly fewer PCV-infected *Cx. annulirostris* were infected or transmitted WNV compared to PCV negative mosquitoes. Immunohistochemistry revealed that PCV localized in the midgut epithelial cells, which are the first site of infection with WNV.

**Conclusions:**

Our results indicate that PCV cannot infect *Cx. annulirostris* via the oral route, nor be transmitted in saliva or vertically to progeny. We also provide further evidence that prior infection with insect-specific viruses can regulate the infection and transmission of pathogenic arboviruses.

## Background

Insect-specific flaviviruses (ISFs) have been isolated from numerous species of mosquitoes from different genera, and from most regions of the world (reviewed in [1]). These flaviviruses differ from medically important members of the genus, such as West Nile virus (WNV), dengue viruses (DENVs) and Zika virus (ZIKV), in that they do not replicate in vertebrate hosts and are transmitted directly between mosquitoes [2]. Laboratory studies on a limited number of ISFs suggest they are maintained in mosquito populations by vertical transmission from the infected female to her progeny via infected eggs [3]. Preliminary findings that natural regulation of the transmission of pathogenic arboviruses, such as WNV, may also occur in mosquitoes persistently infected with ISFs has created intense interest in the role of ISFs in the ecology and epidemiology of vector-borne viral diseases [3–6].

Palm Creek virus (PCV) was the first ISF to be discovered in Australia, where it was isolated from *Coquillettidia xanthogaster* mosquitoes, captured from northern Australia [4, 7]. Although this mosquito species is susceptible to infection with WNV and Ross River virus (RRV), a prevalent alphavirus in Australia, it is not considered to be a major arbovirus vector [8, 9]. It was subsequently found that PCV was most closely related to Nakiwogo virus, an ISF isolated from *Mansonia* species in Uganda, and clustered more broadly with *Culex*-associated ISFs, such as *Culex flavivirus* (CxFV) [4].

Earlier *in vitro* studies revealed that *Aedes albopictus* (C6/36) cells, previously infected with PCV, were significantly less permissive to WNV and Murray Valley encephalitis virus (MVEV) infection and replication, when compared to WNV or MVEV-only infected cells, suggesting that PCV interfered with infection and/or replication of the vertebrate-pathogenic virus [4]. Furthermore, since prior infection with PCV failed to alter the replication of the alphavirus RRV in C6/36 cells, this effect appeared to be flavivirus-specific. Similar findings have subsequently been reported for other ISFs [5].

In the current study we extended the *in vitro* experiments of Hobson-Peters et al. [4] by investigating the effect of PCV on the replication and transmission of WNV in the mosquito *Culex annulirostris,* the primary Australian vector of encephalitic flaviviruses, including WNV and MVEV. To facilitate this, we characterized different routes of PCV infection and transmission by *Cx. annulirostris* by exposing mosquitoes to virus via an infectious blood meal or intrathoracic inoculation, before assessing their ability to transmit the virus horizontally in saliva or vertically to progeny. The ability for PCV to infect and to be transmitted by other mosquito genera was also examined in *Ae. aegypti* and *Aedes vigilax*, which are major DENV and RRV vectors, respectively [10].

## Methods

### Viruses

The PCV strain was isolated from Palm Creek, near Darwin, Australia in 2010. It had been passaged 4 times in C6/36 cells. Two strains of the WNV Kunjin subtype were examined in the superinfection experiments. The strain WNV_KUNMRM16_ was isolated from *Cx. annulirostris* from Kowanyama in 1960 and had been passaged an unknown number of times in C6/36 cells. The WNV_KUN2009_ strain was originally isolated from *Cx. annulirostris* collected from Kununurra, Western Australia, in 2009, and had been passaged twice in C6/36 cells, and once in porcine stable equine kidney (PSEK) cells before a final passage in C6/36 cells.

### Mosquitoes

Colonized *Cx. annulirostris* were obtained from a colony housed at the Australian Army Malaria Institute, Brisbane, Australia. This colony was established from mosquitoes collected from the Boondall Wetlands near Brisbane in 1998 and had been in colony for over 50 generations. Unless otherwise stated, experiments with *Cx. annulirostris* were undertaken using colonized mosquitoes. However, due to a shortage of colonized *Cx. annulirostris*, we performed some of the PCV/WNV interaction experiments and vertical transmission experiments using field collected mosquitoes. Field populations of *Cx. annulirostris* were collected using CO_2_-baited Centers for Disease Control light traps (Model 512, John Hock Co., Gainesville, Florida) from the suburbs of Hemmant and Tingalpa, Brisbane. Adults from field collections were used for the vertical transmission experiments. Progeny from these field populations were also obtained using the protocol of van den Hurk et al. [11], with the exception that an anaesthetized mouse instead of a rat was used as a blood meal source. The use of animals was approved by Forensic and Scientific Services Animal Ethics Committee (approval number 11P02). The ability for PCV to infect other mosquito genera was assessed using *Ae. aegypti* and *Ae. vigilax*, which were obtained from Rockhampton, Australia, and Hemmant, respectively. The *Ae. aegypti* were in the F_1_ generation, whilst *Ae. vigilax* were F_0_ progeny from the original field collections.

### Modes of transmission

#### Oral exposure

To explore whether *Cx. annulirostris* could be infected with PCV by the oral route, 5–7 day old females, that had been starved for 18 h, were exposed to cotton pledgets [12] soaked with a blood/virus mixture. This mixture consisted of washed defibrinated sheep blood (Applied Biological Products Management – Australia, Aldinga Beach, South Australia), 1 % sugar and PCV to provide a final titer of 10^5^ tissue culture infectious dose (TCID)_50_/ml). To confirm this virus titer during feeding, pre- and post- feeding samples of blood/virus mixture were diluted 1:10 in growth medium (GM; Opti-MEM, GIBCO, Life Technologies, Grand Island, NY USA), supplemented with 3 % foetal bovine serum (FBS; *In Vitro* Technologies, Australian origin), antibiotics and antimycotics (GIBCO, Life Technologies, Grand Island, NY USA) and stored at -80 °C. The following day, mosquitoes were briefly anaesthetized with CO_2_ and blood engorged mosquitoes were transferred into 900 ml gauze covered containers. Mosquitoes were incubated at 28 °C with 12:12 light:dark (L:D) and high relative humidity with 10 % sucrose as food source. After an incubation period of 16 days, the body, legs + wings and saliva expectorates from each mosquito were collected separately to assess infection, dissemination and transmission. For transmission, saliva expectorates were collected using the method of Aitken [13]. Briefly, legs + wings were removed and the proboscis of the mosquito inserted into a capillary tube containing GM with 20 % FBS. After 20–30 min, the contents of the capillary tube were expelled into 600 μl of GM + 3 % FBS. The body and legs + wings were separately placed into 2 ml U-bottom tubes containing 1 ml of GM + 3 % FBS and one 5 mm stainless steel bead. All samples were stored at -80 °C until further processing.

#### Intrathoracic inoculation

Next, we examined whether *Cx. annulirostris*, *Ae. aegypti* and *Ae. vigilax* could be infected via intrathoracic inoculation [14], which circumvents the midgut infection and escape barriers, and allows a standard amount of virus to be delivered. For these injections, CO_2_ anaesthetized mosquitoes were injected on a refrigerated table with 200 nl of virus diluted in GM + 3 % FBS to provide a final titer of approximately 10^5^ TCID_50_/ml. Bodies, legs + wings and saliva expectorates were collected as described above on days 10, 14 and 16 following injection, for *Ae. vigilax*, *Ae. aegypti* and *Cx. annulirostris,* respectively, and stored at -80 °C. To determine the tissue tropism of PCV following intrathoracic inoculation, a subset of *Cx. annulirostris* with their legs, wings and antennae removed, were fixed in 10 % neutral-buffered formaldehyde for 24 h, before being stored in 70 % ethanol until processed for immunohistochemistry (IHC).

#### Vertical transmission

Field-collected *Cx. annulirostris* adults were inoculated intrathoracically with 10^4.7^ TCID_50_/ml of PCV. Following inoculation, mosquitoes were maintained in 30 × 30 × 30 cm cages (BugDorm, MegaView Science Co., Ltd, Taiwan) at 28 °C, 12:12 L:D and high relative humidity with 15 % honey water as a food source. Mosquitoes were daily offered a blood meal of defibrinated sheep blood via a Hemotek feeding apparatus (Discovery Workshops, Accrington, Lancashire, UK) fitted with a pig intestine membrane. An oviposition container containing 250 ml of ddH_2_O was added to each cage. Egg rafts were harvested daily and placed separately in 900 ml containers containing 400 ml of ddH_2_O. First- and second-instar larvae were fed a slurry of Tropical Fish flakes (Wardley’s Tropical Fish Food Flakes, The Hartz Mountain Corporation, New Jersey) mixed half/half with brewer’s yeast (Brewer’s Yeast, Healthy Life), whilst third- and fourth-instar larvae were fed on cichlid pellets (Kyorin Co. Ltd, Himeji, Japan). Pupae were transferred into a 150 ml cup within 900 ml gauze-covered containers and maintained as described above. After 7 days, mosquitoes were killed by CO_2_ gas, and males and females placed in pools of 10 before being stored at -80 °C.

### Effect of PCV infection on WNV replication and transmission

To assess the effect of prior PCV infection on subsequent WNV replication and transmission, field collected or colonized *Cx. annulirostris* were injected with 200 nl of a 10^4.0^TCID_50_/ml dose of PCV. Another cohort was injected with GM + 3 % FBS only. After 7–8 days incubation at 28 °C, 12:12 L:D and high relative humidity, PCV-inoculated and PCV negative mosquitoes were offered blood meals containing 10^7^ TCID_50_/ml of WNV. In two separate experiments, colonized *Cx. annulirostris* were offered WNV_KUNMRM16_ via blood soaked pledgets, whilst females reared from field-collected *Cx. annulirostris* were exposed to WNV_KUN2009_ via hanging drops [15]. The reason for implementing a different oral feeding method for the field populations was insufficient feeding rates of < 5 % with the pledgets. Also, unexpectedly low WNV_KUNVMRM16_ infection rates (< 4 %) in the PCV negative mosquitoes compromised our ability to draw any meaningful conclusions from the results of the blood feed involving this virus strain. Thus, we excluded the results of this experiment from the current paper. Colonized *Cx. annulirostris* were exposed to WNV via intrathoracic inoculation with 200 nl of 10^5.0^ TCID_50_/ml of WNV_KUNMRM16_ or 10^5.7^ TCID_50_/ml of WNV_KUN2009_. Ten to 12 days after exposure to WNV, bodies and saliva expectorates were harvested as described above and stored at -80 °C. For IHC, additional WNV_KUN2009_-inoculated mosquitoes were fixed and stored as described above.

### Virus assays

#### Cell culture enzyme immunoassay

Pre- and post-feeding blood samples and inoculum at the beginning and end of injection period were inoculated as 10-fold dilutions in the wells of a 96-well microtiter plate seeded with C6/36 cells. Plates were incubated at 28 °C for 7 days before being fixed with 20 % acetone and stored at -20 °C. The bodies and legs + wings from all experiments, and females and males collected in pools from the vertical transmission experiment were homogenized in a QIAGEN TissueLyser II (Qiagen, Hilden, Germany), centrifuged at 14,000 *g* before being filtered through a 0.2 μm filter (Pall Corporation, Ann Arbor, MI). The body filtrate and filtered saliva expectorates were inoculated as 10-fold dilutions in the wells of a 96-well microtiter plate containing confluent monolayers of C6/36 cells. Legs + wings and mosquito pools were inoculated in quadruplicate onto C6/36 cell monolayers within a 96-well microtiter plate. Plates were incubated, fixed and stored as described above. Presence of virus in fixed plates was determined by fixed cell enzyme immunoassay using specific monoclonal antibodies 3D6 for PCV [4] and 4G2 for WNV [16].

#### Detection of PCV RNA in mosquito pools

Nucleic acids were extracted from the pools of *Cx. annulirostris* progeny using the Qiagen BioRobot Universal System and QIAamp Virus BioRobot MDx Kit (Qiagen, Clifton Hill, Australia). PCV RNA was amplified using primers designed in this study to NS2A of the PCV56 polyprotein (PCV F1: GGA GAG TTC GAG AGG AGT GAG C and PCV R1: CAA CTG GGC AAT CAG ATG TGC). Amplification was performed using the Superscript III One-Step RT-PCR System with Platinum Taq DNA polymerase (Invitrogen) with the following RT-PCR cycling conditions: RT for one cycle each at 45 °C/30 min and 94 °C/2 min, and then PCR for 50 cycles at 94 °C/30 s, 69 °C/30 s, and 68 °C/30 s.

#### Immunohistochemical detection of PCV in situ in mosquitoes

Mosquitoes were mounted in paraffin blocks for histology and IHC. Five micrometer serial sections were collected on Superfrost PLUS pre-cleaned microscope slides (MenzelGlӓser, Braunschweig, Germany), heat-treated (2 h, 60 °C), and deparaffinized through a xylene-ethanol series. The sections were subjected to antigen retrieval by heating in either citrate-buffer, pH 6.0 for PCV-detection or EDTA-buffer, pH 9.0 for WNV detection (Target Retrieval™, DAKO corp., Carpentaria, CA) for 25 min at 96 °C followed by 20–25 min cooling to room temperature. This was followed by three blocking steps (0.3 % hydrogen peroxide in water for 10 min, 0.15 M glycine in PBS for 10 min, DAKO blocking agent for 30 min) at room temperature with brief rinses with Tris-buffered saline/Tween-20 (TBST) in between. WNV antigen detection was performed using the NS1-specific mAb 4G4 [17] with incubation at room temperature for 2 h, while for PCV-antigen detection mouse anti-PCV hyper-immune serum was applied (1:400 dilution in DAKO-blocking solution) and slides incubated overnight (16–18 h) at 4 °C. This was followed by multiple rinses with TBST over a period of 10–15 min. Antibody binding was visualized using the mouse Envision-kit from DAKO and the chromogen amino-ethylcarbazole (AEC) resulting in a red signal. The sections were counterstained with Mayer’s hematoxyline and mounted with DAKO Paramount agent. Sections were examined on a Nikon Eclipse 50i microscope and digital microphotographs captured with a Nikon DS-Fi1 camera with a DS-U2 unit and NIS elements F software, and are presented without further manipulation.

#### Kinetics of viral replication in vitro

Two cell lines were used in these experiments: C6/36 cells, which are deficient in the siRNA response due to a point mutation in Dicer-2, and RML-12 (*Ae. albopictus*) cells, which have a fully functional Dicer-2-dependent RNAi response [7]. The C6/36 cells were cultured in RPMI 1640 medium supplemented with 5 % FBS, whilst RML-12 cells were cultured in Leibovitz's L-15 medium supplemented with 10 % FBS and 10 % tryptose phosphate buffer. The C6/36 and RML-12 cells were seeded at a density of 1 × 10^5^. Monolayers were inoculated in triplicate at a multiplicity of infection (MOI) of 0.1 with PCV or WNV_KUNMRM16_. After incubation at 28 °C for 1 h, the inoculum was removed and wells were washed three times with sterile PBS with fresh cell-specific media added for further incubation at 28 °C. Supernatant was harvested at the hours 2, 24, 48, 72, 96, 120, 144 and 168. Infective viral titers from each time-point were determined as described above.

### Analyses

The titer of the blood/virus suspension, and the mosquito bodies and saliva expectorates was calculated using the method of Reed and Meunch [18] and expressed as TCID_50_/ml. Differences in PCV body titer between the three mosquito species was analysed using a Kruskill-Wallis test, with a Dunn’s post hoc multiple comparisons test. WNV infection, dissemination and transmission rates in PCV infected and non-infected *Cx. annulirostris* were compared using Fisher’s exact tests. Differences in WNV titer within bodies and saliva expectorates of PCV infected and non-infected *Cx. annulirostris* were analyzed using Mann-Whitney U tests. Replication of PCV and WNV_KUNMRM16_ in the C6/36 and RML-12 cells was compared using a two-way ANOVA. All statistical tests were performed using Graphpad Prism statistical software Version 6 (GraphPad Software, Inc, San Diego, USA).

## Results

### Mode of PCV infection and transmission

**Fig. 1:**
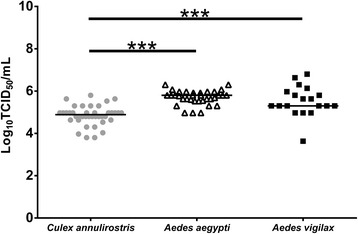
Viral titers in the bodies of *Culex annulirostris, Aedes aegypti* and *Aedes vigilax* mosquitoes injected intrathoracically with PCV. Each point on the plot represents an individual infected mosquito and bars denote medians. *P* < 0.001 (***), Kruskill-Wallis test, with a Dunn’s *post-hoc* multiple comparisons test. The comparison between *Ae. aegypti* and *Ae. vigilax* was non-significant (*P* > 0.05)

None of 44 *Cx annulirostris* were infected with PCV after imbibing an infectious blood meal containing 10^5^ TCID_50_/ml of virus. In contrast, 100 % (53/53) of *Cx. annulirostris*, 100 % (34/34) of *Ae. aegypti* and 95 % of *Ae. vigilax* (19/20) were infected after being intrathoracically inoculated with PCV. Mean body titers varied significantly (*P* < 0.001) between the three species (Fig. 1). PCV was not detected in the saliva of any infected mosquitoes.

In the vertical transmission experiments, a total of 1038 progeny from 59 PCV infected *Cx. annulirostris* were collected, comprising 587 females and 451 males. No PCV RNA was detected in any of the pools of female or male *Cx. annulirostris* tested.

### Effect of PCV infection on WNV replication and transmission in *Cx. annulirostris*

#### Oral exposure

**Fig. 2:**
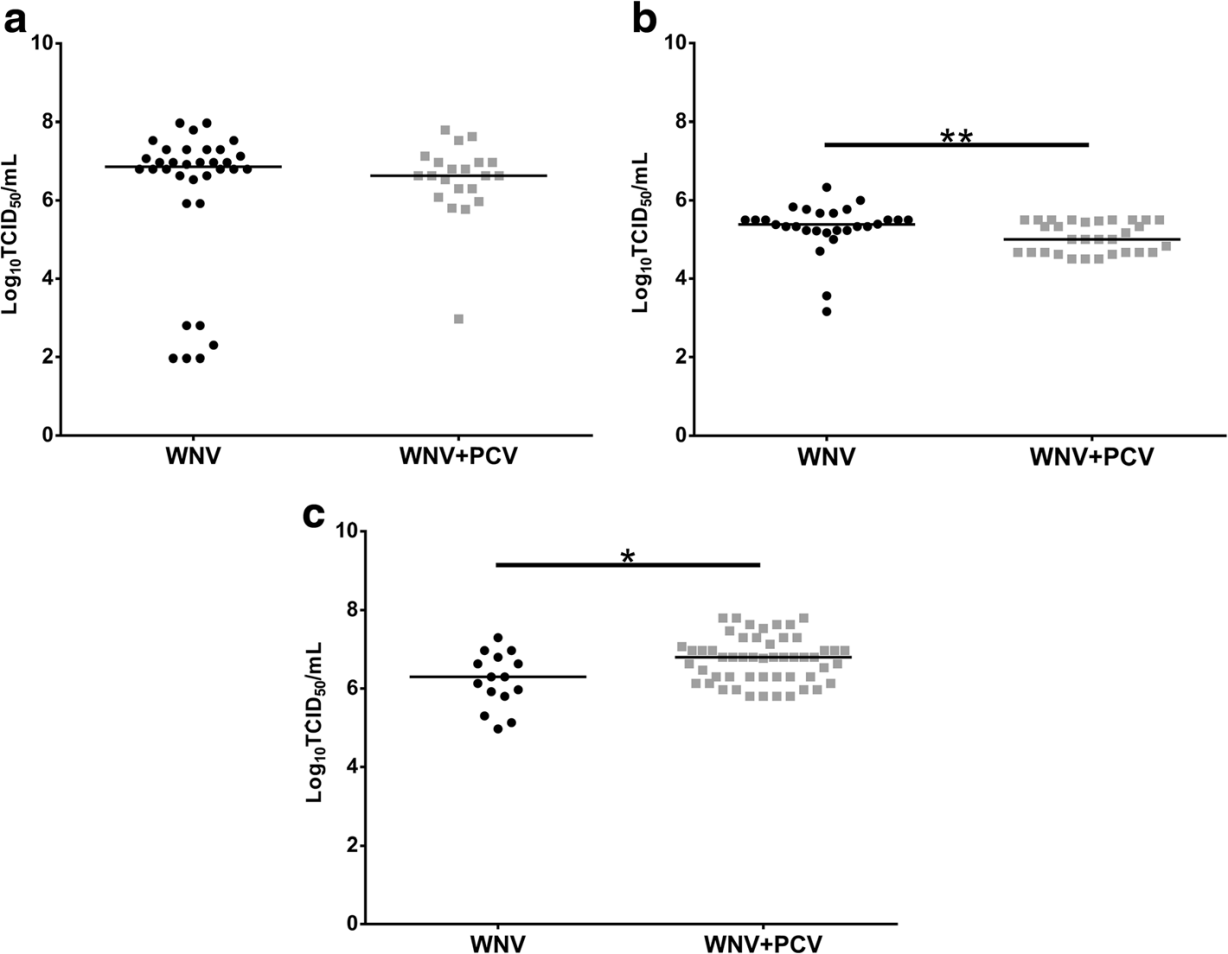
Impact of PCV on WNV replication in bodies of *Cx. annulirostris* 10–12 days after being exposed to WNV_KUN2009_ via ingestion of an infectious blood meal (**a**) or intrathoracic inoculation (**b**), or WNV_KUNMRM16_ via intrathoracic inoculation (**c**). Each point on the plot represents an individual infected mosquito and bars denote medians. *P* < 0.01 (**), *P* < 0.05 (*) Mann-Whitney *U* test

**Fig. 3:**
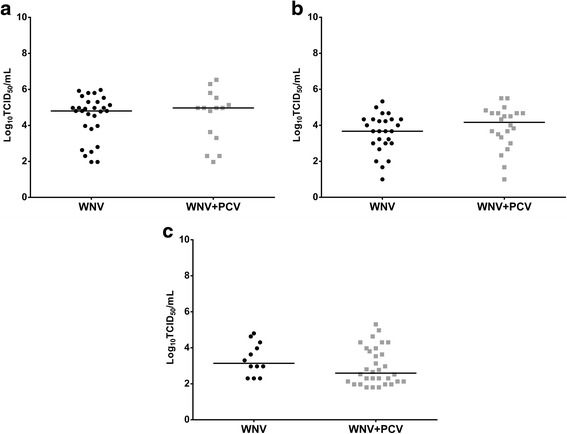
Impact of PCV on WNV replication in saliva of *Cx. annulirostris* 10–12 days after being exposed to WNV_KUN2009_ via ingestion of an infectious blood meal (**a**) or intrathoracic inoculation (**b**), or WNV_KUNMRM16_ via intrathoracic inoculation (**c**). Each point on the plot represents an individual infected mosquito and bars denote medians

Following feeding on a blood meal containing 10^7^ TCID_50_/ml of WNV_KUN2009_, the infection rate in PCV positive *Cx. annulirostris* was significantly lower (*P* = 0.025) than in PCV negative *Cx. annulirostris* (Table 1). Dissemination rates as evidenced by recovery of virus from the legs + wings were also lower in PCV positive versus PCV negative *Cx. annulirostris*, although the difference was not significant (*P* > 0.05). Finally, significantly fewer (*P* = 0.017) PCV infected *Cx. annulirostris* transmitted WNV_KUNV2009_ compared to PCV negative *Cx. annulirostris*. When those mosquitoes with a disseminated infection were analyzed, significantly fewer (*P* = 0.049) PCV infected *Cx. annulirostris* transmitted WNV_KUNV2009_ compared to PCV negative *Cx. annulirostris*. Despite significant differences in infection and transmission rates, there was no significant difference (*P* > 0.05) in WNV titers in bodies and saliva expectorates between PCV positive and negative *Cx. annulirostris* (Figs. 2a and 3a).

**Table 1:** Infection, dissemination and transmission rates in *Cx. annulirostris* after exposure to WNV by either oral route or intrathoracic inoculation (IT). Mosquitoes previously infected with PCV were compared to mosquitoes mock infected with growth medium

	Infection^a^				Dissemination^b^				Transmission^c^				Transmission/Dissemination^d^			
Mode of exposure (virus strain)	WNV		WNV + PCV		WNV		WNV + PCV		WNV		WNV + PCV		WNV		WNV + PCV	
Oral (WNV_KUN2009_)	76	(34/45)	51	(21/41)^e^	69	(31/45)	51	(21/41)	64	(29/45)	37	(15/41)^e^	94	(29/31)	71	(15/21)^e^
IT (WNV_KUN2009_)	93	(28/30)	100	(30/30)	93	(28/30)	93	(28/30)	90	(27/30)	73	(22/30)	96	(27/28)	79	(22/28)
IT (WNV_KUNMRM16_)	88	(15/17)	100	(52/52)	Not tested				80	(12/15)	62	(35/52)	Not tested			

^a^Percentage of mosquitoes containing virus in their bodies (number positive/number tested)

^b^Percentage of mosquitoes containing virus in their legs and wings (number positive/number tested)

^c^Percentage of mosquitoes containing virus in the saliva expectorates (number positive/number tested)

^d^Percentage of mosquitoes with a disseminated infection containing virus in the saliva expectorates (number positive/number disseminated)

^e^Fisher’s exact test *P*-value < 0.05 for comparisons between *Cx. annulirostris* infected with WNV only and those infected with WNV and PCV

#### Intrathoracic inoculation

Irrespective of the WNV strain, there was no significant difference (*P* > 0.05) in WNV infection rates between PCV positive and negative *Cx. annulirostris* (Table 1). Although WNV transmission rates were lower in PCV infected mosquitoes compared to mosquitoes without PCV, this difference was not significant (*P* > 0.05).

There did not appear to be a consistent pattern in the replication of the two WNV strains in the PCV infected and PCV negative *Cx. annulirostris* (Figs. 2b, c, and 3b, c). Interestingly, PCV infected bodies had a significantly higher (*P* = 0.004) WNV_KUNMRM16_ titer than those without PCV. Conversely, mosquitoes without PCV infection had significantly higher (*P* = 0.007) WNV_KUN2009_ body titers than PCV infected *Cx. annulirostris*. There was no significant difference (*P* > 0.05) in WNV saliva titers between the PCV positive and negative *Cx. annulirostris*, irrespective of the WNV strain tested.

### Immunohistochemical detection of PCV in situ in mosquitoes

**Fig. 4:**
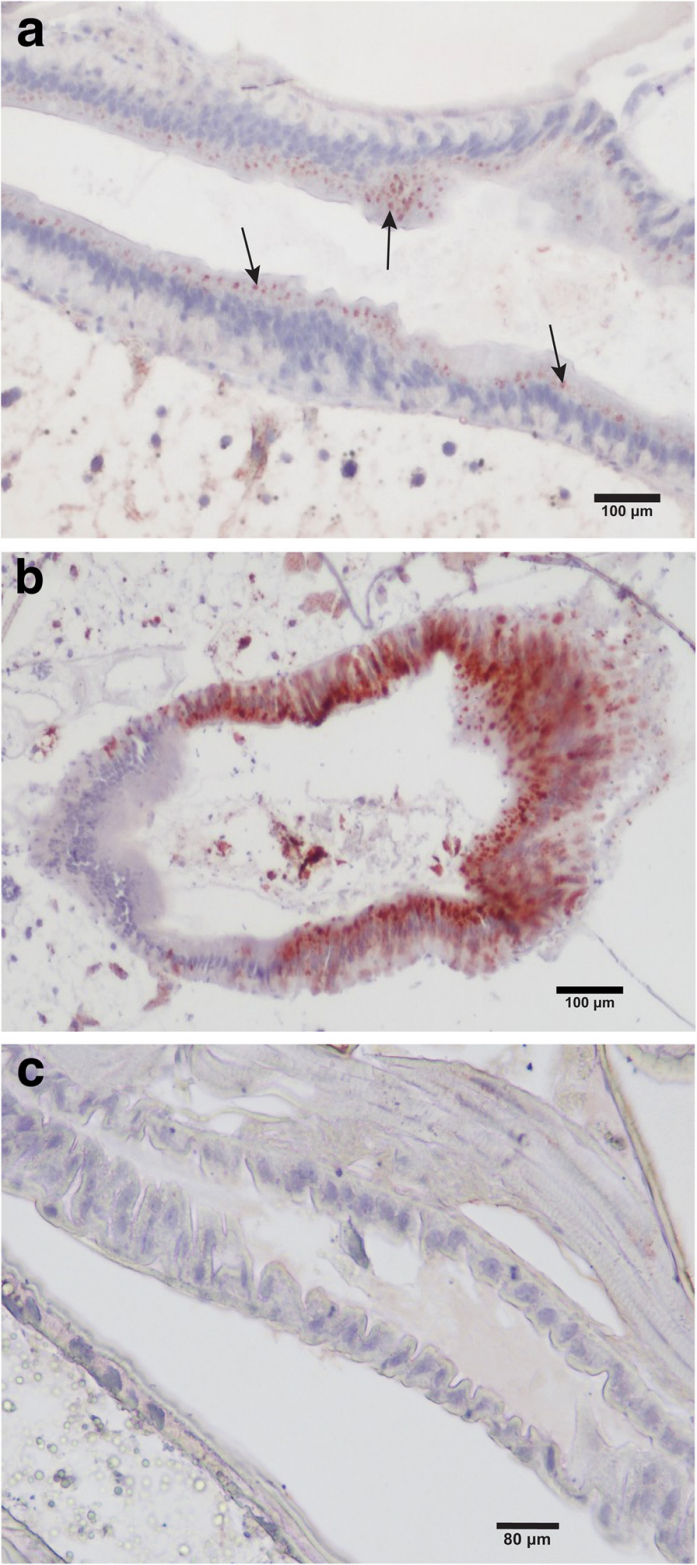
Immunohistochemical detection of PCV (**a**) and WNV (**b**) in midgut epithelial cells (red signal) of infected female *Cx. annulirostris* mosquitoes. **c** antibody isotype control. Hematoxylin was used as the counterstain

To understand the nature of the PCV-mediated exclusion of WNV from mosquitoes orally infected with the virus, we examined the tissue tropism of PCV in *Cx. annulirostris* mosquitoes seven days after intrathoracic inoculation. After fixation in formalin and thin sectioning for IHC, staining of infected mosquito sections (*n* = 14) with PCV-specific antiserum revealed that the virus specifically localized in epithelial cells lining the midgut (Fig. 4a) and was not present in any other tissues. In contrast, WNV infection in mosquitoes (*n* = 4) occurred in most tissues and organs, including the midgut (Fig. 4b), salivary glands and neural tissues including eyes (data not shown).

### Comparative growth kinetics of PCV and WNV in mosquito cells

**Fig. 5:**
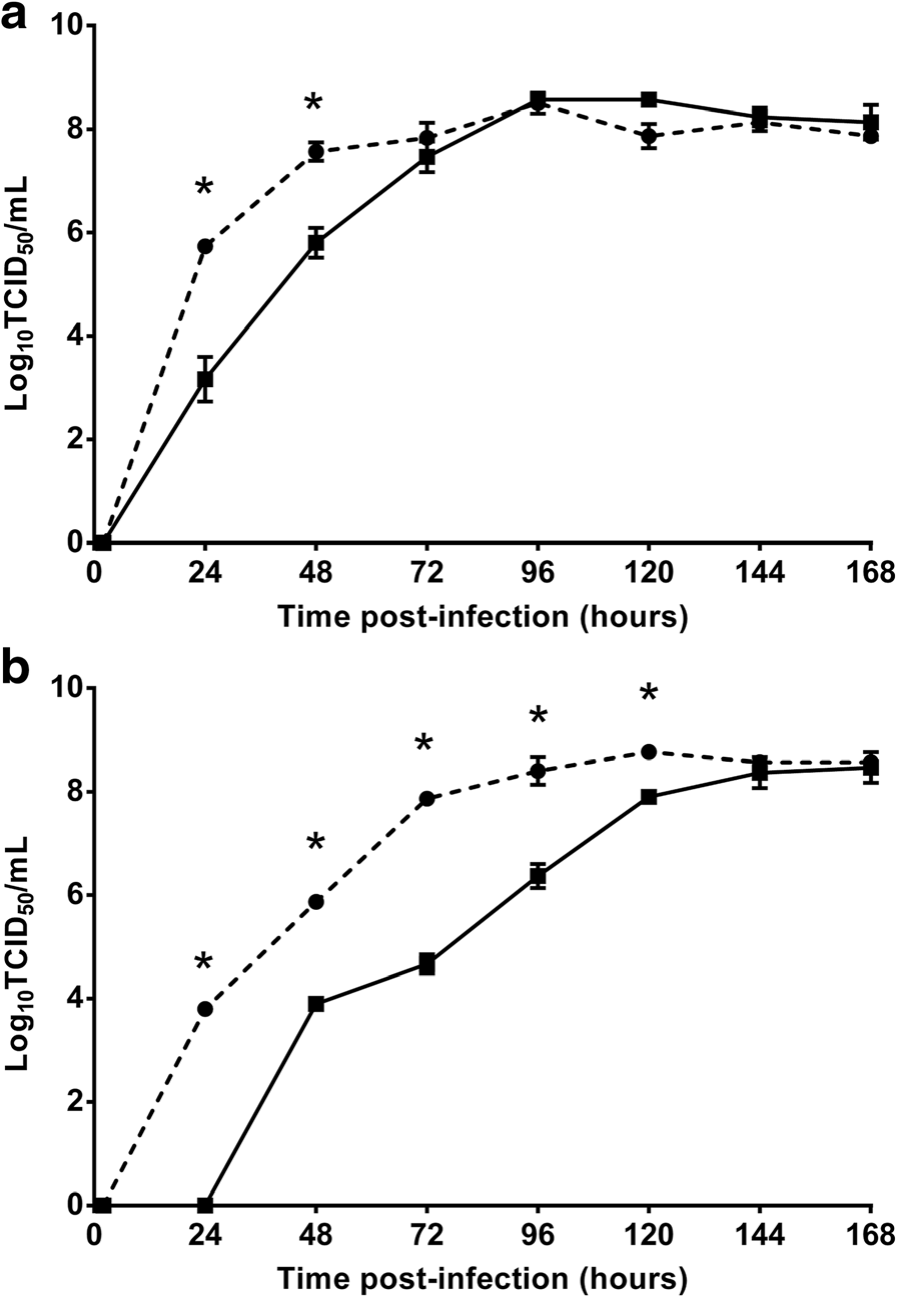
Comparative growth kinetics of PCV (circles) and WNV (squares) in C6/36 (RNAi-deficient) (**a**) and RML-12 (RNAi-competent) (**b**) *Aedes albopictus* cells. Cells were infected with either PCV or WNV at an MOI of 0.1 and infectious titers at each time-point (up to 5 or 7 days) determined by titration of culture supernatant on C6/36 cells and detection of infected wells by fixed cell ELISA. Error bars represent standard deviation and asterisks indicate significance (*P* < 0.0001) as determined by a two-way ANOVA

To compare the efficiency of replication of PCV and WNV in mosquito cells, the rate of growth of these viruses in C6/36 cells (deficient siRNA response) and RML-12 cells (competent siRNA response) was assessed. In both cell lines, PCV replicated more rapidly than WNV during the first 72 h, with titers 100–1000 fold higher between 24 and 48 h (Fig. 5). By 96 h the peak titers were similar for both viruses in C6/36 cells (~10^8^ infectious units per ml; Fig. 5a). While a similar titer was reached for both viruses in RML12 cells, PCV levels remained significantly higher than those of WNV until the 144 h time point (Fig. 5b). A comparison between growth rates of PCV in both cell lines further revealed that C6/36 cells (Fig. 5a) yielded approximately 10-fold higher titers than RML-12 cells (Fig. 5b) at 24 (*P* < 0.05) and 48 h (*P* < 0.05), with similar titers reached by 72 h. A similar trend was seen for WNV with titers lagging in RML-12 s by 10–100 fold until the 120 h time point (Fig. 5).

## Discussion

In this study we assessed modes of transmission of PCV, the prototype insect-specific flavivirus in Australia, and its ability to modulate replication and transmission of WNV. Although *Cq. xanthogaster* has yielded all isolates of PCV to date [4, 7], this species is difficult to colonize in the lab and could not be collected in sufficient numbers from the field to undertake laboratory experiments. Therefore, *Cx. annulirostris, Ae. aegypti* and *Ae. vigilax*, which are common Australian species and known vectors of a range of arboviruses, were used for laboratory infection studies. We predominantly focused on *Cx. annulirostris*, because this species is the key vector of the encephalitic flaviviruses WNV and MVEV, and Hobson-Peters et al. [4] conducted their *in vitro* experiments with these viruses. Furthermore, PCV RNA was detected in *Cx. annulirostris* collected during the original study when the virus was first identified, although the virus was not subsequently isolated from these mosquitoes (R. A. Hall and J. Hobson-Peters, unpublished data).

Our experiments demonstrated that PCV could not infect *Cx. annulirostris* by the oral route using a biologically relevant dose. However, the virus could replicate efficiently in this species, as well as in *Ae. aegypti* and *Ae. vigilax*, if injected intrathoracically. Post-infection, the virus could not be detected in the saliva or progeny collected from any of the PCV-infected mosquitoes. This suggests that the virus is not transmitted horizontally or vertically by these species. Even though the sample sizes of mosquitoes are comparable to previous studies [3, 19], the lack of evidence of horizontal or vertical transmission in the current study may highlight the narrow host range of PCV, in that infection is restricted primarily to *Cq. xanthogaster*. Alternatively, it could indicate that the mode of infection of the female parent mosquito is important. For instance, *Cx. pipiens* inoculated intrathoracically with CxFV could not transmit the virus vertically, whereas 100 % of naturally infected females transmitted the virus to their progeny [19].

The study by Hobson-Peters et al. [4] showed that mosquito cells previously infected with PCV were less permissive than uninfected cells to replication of WNV or MVEV, indicating an ISF-induced mechanism of interference or super infection exclusion. Indeed, these findings and results of other studies [3, 6] suggested that ISFs may suppress the replication of heterologous flaviviruses in mosquitoes and potentially regulate their transmission. In the present study we further explored this hypothesis *in vivo*, using sequential infection of laboratory mosquitoes. As predicted by the *in vitro* data, the mosquitoes previously infected with PCV were less susceptible to oral infection with WNV and less competent to transmit the virus when compared to uninfected mosquitoes. However, this phenomenon was dependent on the mode of the secondary infection, with WNV infection and transmission rates after intrathoracic injection relatively unaffected by prior PCV infection of the mosquito.

The vector competence of mosquitoes for arboviruses is influenced by a number of factors, including genetics of the mosquito population, virus strain, environmental conditions or the presence of endosymbionts which can impact infection [20, 21]. Despite being a pest species in some areas [9], *Cq. xanthogaster* is not considered to be an important arbovirus vector in Australia and has not yielded any isolates of WNV or MVEV. This is despite a large number of pools of this species being processed during periods when these viruses have been isolated from recognized vectors [22, 23]. Thus, the minor role that *Cq. xanthogaste*r serves as an arbovirus vector may be explained by inhibition of pathogenic viruses caused by systemic infection with PCV, which can be prevalent in some populations of this species [4]. However, similar laboratory studies to the experiments described herein need to be conducted with *Cq. xanthogaste*r to confirm this hypothesis.

The above findings are also consistent with our IHC data that showed specific localization of PCV to the epithelial cells lining the midgut of *Cx. annulirostris* after intrathoracic inoculation. Indeed, these cells are the site of entry of WNV infection following ingestion of an infectious blood meal [24]. This suggests that prior infection of the midgut epithelial cells with PCV may inhibit their infection with WNV. The precise mechanism for this phenomenon could involve competition between PCV and WNV for cellular resources required for efficient replication. Indeed, our *in vitro* growth kinetics data suggest that in some mosquito cells, PCV replicates more efficiently than WNV during the early stages of infection. This may provide an advantage to PCV over WNV for replication in co-infected cells of the midgut. Alternatively, the upregulation of antiviral responses in the mosquito by PCV infection, including RNAi pathways (siRNA and piRNA) and Janus kinase (Jak)-signal transducer and activator of transcription (STAT) (Jak-STAT) activation via Vago, may also affect WNV replication upon subsequent infection [25–29].

## Conclusions

The results of our experiments are consistent with previous studies that show prior infection of *Culex* mosquitoes with the insect-specific flaviviruses CxFV or Nhumirim virus, significantly suppressed or delayed WNV transmission by some species [3, 6]. Future studies should also investigate whether prior infection with *Aedes* ISFs such as CFAV, Kamiti River virus or another Australian ISF, Parramatta River virus (PaRV) [7, 30, 31], have a similar effect on the transmission of DENV and ZIKV by *Ae. aegypti* and/or *Ae. albopictus*, which are the primary vectors of these pathogenic flaviviruses*.* Although intrathoracic inoculation was the only mode of infection of *Cx. annulirostris* with PCV, the detection of PCV antigen in the epithelial cells of the midgut of this species was consistent with recent IHC studies on PaRV (B. McLean and H. Bielefeldt-Ohmann, unpublished data). In these studies, PaRV was visualised in F_1_*Ae. vigilax* reared from field collected adults, suggesting natural vertical transmission. Thus, the tissue tropism in the midgut epithelial cells of mosquitoes appears similar within these two ISFs, despite the different modes of infection. Together, this provides evidence that exclusion of the secondary virus could be due to the presence of the ISF in the midgut epithelial cells, which are the first site of virus binding and infection in the mosquito. Further investigation of the mechanisms of infection and replication of ISFs within their natural vectors will provide valuable information about the ability for ISFs to regulate the transmission of pathogenic flaviviruses and the mechanisms which facilitate this phenomenon.

## Abbreviations

AEC, amino-ethylcarbazole; ANOVA, analysis of variance; CO_2_, carbon dioxide; CxFV, *Culex* flavivirus; DENVs, dengue viruses; EDTA, ethylenediaminetetraacetic acid; FBS, fetal bovine serum; GM, growth media; IHC, immunohistochemistry; ISF, insect specific flavivirus; Jak-STAT, Janus kinase-signal transducer and activator of transcription; MOI, multiplicity of infection; MVEV, Murray Valley encephalitis virus; NS, non-structural protein; PaRV, Parramatta River virus; PBS, phosphate buffered saline; PCV, Palm Creek virus; piRNA, piwi-interacting ribonucleic acid; RNA, ribonucleic acid; RNAi, ribonucleic acid interference; RRV, Ross River virus; RT-PCR, reverse transcriptase-polymerase chain reaction; siRNA, small interfering ribonucleic acid; TBST, Tris-buffered saline/Tween-20; TCID, tissue culture infectious dose; WNV, West Nile virus; WNV_KUN_, West Nile virus Kunjin subtype; ZIKV, Zika virus.
